# Refining thresholds and timing in V/Q matching assessment during prone positioning in ARDS

**DOI:** 10.1186/s13613-025-01530-3

**Published:** 2025-07-26

**Authors:** Nan Xiong, Yinde Huang

**Affiliations:** 1 Department of Gastroenterological Surgery, Peking University People’s Hospital, Beijing, 100044 China; 2 Department of Breast and Thyroid Surgery, Chongqing General Hospital, Chongqing, 401147 China

## To the Editor,

We read with great interest the recent article by Wang et al. reporting a two-center prospective study investigating the association between early prone positioning (PP)-induced improvement in ventilation-perfusion (V/Q) matching and ICU mortality in patients with moderate to severe ARDS [1]. Their use of electrical impedance tomography (EIT) to noninvasively assess regional lung function adds valuable insight into physiologic responsiveness to PP. However, we would like to offer two methodological considerations that may further enhance the clinical applicability of their findings.

First, the authors classify patients as V/Q “responders” based on a ≥ 10% improvement in V/Q matching within 4 h of initial PP. This threshold—though grounded in prior small-scale observational studies [2]—is applied without evidence of statistical optimization or physiologic validation in the current cohort. While this threshold builds on prior observational work, further refinement using cohort-specific data—such as spline modeling or ROC curve analysis—could enhance its precision. Exploring V/Q improvement as a continuous predictor may help capture additional prognostic nuance, including patients with sub-threshold but clinically relevant changes. The absence of dose-response modeling (e.g., spline regression or ROC curve analysis) raises the possibility that patients with “sub-threshold” improvements (e.g., 8–9%) may be incorrectly labeled non-responders, despite meaningful physiologic gains. Previous work in ARDS has shown that overly rigid thresholds in variables such as PaO₂/FiO₂ and driving pressure can obscure more nuanced, gradient-dependent risk profiles [3, 4]. A more sophisticated modeling of V/Q improvement, potentially as a continuous covariate in multivariate outcome analysis, may provide a more accurate reflection of its predictive value.

Second, while the study admirably focuses on early V/Q changes (at 4 h post-PP), its central conclusion rests on the implicit assumption that this early improvement reflects a sustained physiologic benefit. However, their own data demonstrate a regression in V/Q matching after returning to the supine position (from 71.2% back down to 61.8%), raising the concern that early gains may be transient. Although early V/Q improvement is encouraging, its durability remains unclear. For example, the observed regression after resupination suggests that a single timepoint may not fully capture long-term benefits. Incorporating repeated assessments could clarify whether early responses translate into sustained physiological advantage. Previous studies suggest that the mortality benefit of PP in ARDS stems not only from transient oxygenation improvement, but from longer-term reductions in ventilator-induced lung injury (VILI) through alveolar recruitment and mechanical homogeneity [5]. As such, evaluating the durability of V/Q improvement—across repeated PP sessions and beyond 4-hour windows—may be essential to determining true treatment responders.

To strengthen the clinical relevance of V/Q-guided stratification, future studies should consider (1) refining responder thresholds using data-driven models; (2) evaluating the time-course and reproducibility of V/Q responses over multiple PP sessions; and (3) integrating additional physiologic and morphologic markers (e.g., lung compliance, EELV, consolidation patterns) into composite predictive frameworks.

The study by Wang et al. represents a valuable step toward physiologically guided stratification in ARDS. Building upon their findings, a more dynamic and continuous evaluation of V/Q changes may further optimize clinical decision-making.

## Electronic supplementary material

Below is the link to the electronic supplementary material.

Supplementary Material 1
