# Supplementary material for: Refining thresholds and timing in V/Q matching assessment during prone positioning in ARDS
Source: Ann Intensive Care. 2025 Jul 26;15:108. doi: 10.1186/s13613-025-01530-3 (PMC12297046; doi:10.1186/s13613-025-01530-3)
Supplement: Supplementary file 1 — Supplementary Material 1 [file 13613_2025_1530_MOESM1_ESM.docx]

Response to Reviewer #1

We sincerely thank Reviewer #1 for the thoughtful and constructive feedback on our Letter entitled “Refining Thresholds and Timing in V/Q Matching Assessment During Prone Positioning in ARDS.” We greatly appreciate the recognition of the methodological considerations raised, and fully agree that the study by Wang et al. offers valuable clinical insight into the prognostic role of early V/Q matching improvement during prone positioning. In response to the reviewer’s suggestion, we have revised the title and softened the tone of our Letter to better reflect our intent—namely, to offer complementary perspectives aimed at enhancing future refinement of V/Q-guided approaches, rather than questioning the demonstrated clinical significance of the authors’ findings. All modifications have been carefully made with the goal of maintaining a constructive and collegial tone.

1. To moderate the tone of our critique and to fully acknowledge the clinical significance of the original study, we have revised the title of our Letter to adopt a more neutral and constructive expression. Revised the title “Refining Thresholds and Timing in V/Q Matching Assessment During Prone Positioning in ARDS”. Highlighted page 1 line 1-2 with red colour.

2. Modified “we would like to offer two methodological considerations that may further enhance the clinical applicability of their findings.”. Highlighted page 1 line 12-13 with red colour.

3. Modified “While this threshold builds on prior observational work, further refinement using cohort-specific data—such as spline modeling or ROC curve analysis—could enhance its precision. Exploring V/Q improvement as a continuous predictor may help capture additional prognostic nuance, including patients with sub-threshold but clinically relevant changes.”. Highlighted page 1 line 18-22 with red colour.

4. Modified “Although early V/Q improvement is encouraging, its durability remains unclear. For example, the observed regression after resupination suggests that a single timepoint may not fully capture long-term benefits. Incorporating repeated assessments could clarify whether early responses translate into sustained physiological advantage.”. Highlighted page 2 line 37-40 with red colour.

5. Modified “The study by Wang et al. represents a valuable step toward physiologically guided stratification in ARDS. Building upon their findings, a more dynamic and continuous evaluation of V/Q changes may further optimize clinical decision-making.”. Highlighted page 2 line 53-55 with red colour.
